# The Treatment of Enuresis.—I

**Published:** 1909-07-24

**Authors:** 


### Diseases of Children.

### THE TREATMENT OF ENURESIS.—I.

In a recent paper* Dr. Leonard Williams has advocated very warmly the administration of thyroid extract in the treatment of enuresis in children. Indeed, from the tone of his communication and the enthusiastic advocacy of the remedy, it might run the danger of being regarded as a specific for this unpleasant and troublesome affection. No doubt many physicians to hospitals for children have made use of the remedy, and obtained good results in suitable cases, namely all those in which there is evidence in favour of a possible defect in thyroid secretion. Such patients are often below the standard in height, weight, general nutrition, and mental capacity, or in one or more of these characteristics. It is noticeable that Dr. Williams found similar evidence in some of his patients, except in so far as the mental capacity was apparently extra good—at any rate, quite up to the average. The dosage was from one to two and a half grains of the extract three times a day. Out of 25 patients thus treated eight were cured, four decidedly better, 13 improved, and only one a complete failure. These results are not very remarkable, for it is well known that in this peculiar affection any systematic course of treatment is likely to cure some cases, probably through the mental effect.

Dr. Williams concludes that adenoids are not the cause of nocturnal enuresis. Probably no one supposes that adenoids are a cause, except in that they may prove the final straw, by leading to imperfect aeration of the blood during sleep and deficient control by the inhibitory cerebral centres. It is well known that the removal of adenoids may be followed by the cessation of enuresis, and that any other operation may have a similar effect. The enuresis may return after a variable period of days or weeks, or it may be started by an operation in a nervous child who has not suffered previously from the affection. Even the psychical effect of a change of surroundings is often enough to stop the affection, perhaps to break the habit with permanently good results. Many a child with enuresis ceases to wet the bed when admitted to hospital. Hence it seems rather unnecessary to consider Dr. Williams' suggestion that possibly the adenoids themselves may be due to thyroid insufficiency, which he regards as *the* cause of enuresis. His argument is still further weakened by his statement that the thyroid secretion "is a regulator of the mechanism by which urinary incontinence is controlled; an excess of secretion being almost, if not quite, as deleterious as an insufficiency." It is an impressive statement, though somewhat vague. Which is the mechanism that is thus controlled? Is it the centre in the lumbar enlargement or inhibitory centres in the higher parts of the central nervous system? Or are we to assume that the efferent and afferent nerves are profoundly affected by a little more or less thyroid extract. Probably the true explanation of the evil effects of excessive dosage is the general disturbance of metabolism and health resulting from its administration. Nevertheless, though much can be said against the theory of thyroid action in these cases, there is no doubt that good results may follow the use of the drug, and it is worthy of trial in selected cases.

The whole treatment of this affection has become complicated by the fact that it is so frequently associated with other affections common in the early years of life. Many of these are merely coincident, whereas others may be indicative of a nervous instability at the bottom of the complaint. It is advisable to remedy any local affection which may be in any way a source of irritation, either of the general health and nervous system or stimulating reflex action. An adherent prepuce or clitoris, phimosis, retained smegma, balanitis and vulvo-vaginitis must be attended to. Such affections constantly exist without causing enuresis, and must not be regarded as primary causes. Remove adenoids and enlarged tonsils if they interfere at all with free breathing, and put the alimentary tract into a healthy state, for there is no doubt that any cause of lowered general health interferes with the cure or maintains the existence of enuresis. On similar grounds threadworms should be got rid of. The internal administration of sulphur lozenges, or a dose of santonin and calomel, may be sufficient for this purpose, and it is even justifiable to give such a dose though there is no evidence that these parasites are present. That they act through reflex irritation of the lumbar centres is unlikely. More probably they are a persistent source of nerve irritation, and lead to an increased excitability of the nervous system as a whole. Similarly it is important to examine the urine. Acquired cases are sometimes found associated with highly acid urine, an excess of uric acid or calcium oxalate crystals, or with bacteriuria. The cure of these conditions may stop the enuresis. Unfortunately comparatively few cases are due to these causes, but they must not be overlooked, for it is useless to treat such patients by a prolonged course of belladonna, for instance, though the patient may get well under such treatment if the urinary abnormality is the effect of temporary causes. Bacteriuria may exist without cystitis. It is more common in girls than boys, because organisms can more readily make their way up the short urethra into the bladder.

No barbarous measures such as corporal punishment, dark cupboards, and bread and water diet should be employed. They are cruel and apt to make a nervous child much worse, though they may occasionally prove beneficial. In the same category may be placed such lines of treatment as the application of nitrate of silver to the neck of the bladder—only in rare instances justifiable—massage of the prostate and neck of the bladder, blistering, and epidural injections of normal saline or one per cent. stovain solution, the effects, if any, being merely psychical.

* *Lancet*, May 1, 1909, p. 1245.

